# Crystal Structures and Molecular Dynamics Simulations of Thermophilic Malate Dehydrogenase Reveal Critical Loop Motion for Co-Substrate Binding

**DOI:** 10.1371/journal.pone.0083091

**Published:** 2013-12-26

**Authors:** Chih-Hung Hung, Tzann-Shun Hwang, Yu-Yung Chang, Huei-Ru Luo, Szu-Pei Wu, Chun-Hua Hsu

**Affiliations:** 1 Department of Agricultural Chemistry, National Taiwan University, Taipei, Taiwan; 2 Graduate Institute of Biotechnology, Chinese Culture University, Taipei, Taiwan; 3 Department of Biotechnology, Yuanpei University, Hsinchu, Taiwan; 4 Genome and Systems Biology Degree Program; Center for Systems Biology, National Taiwan University, Taipei, Taiwan; University of South Florida College of Medicine, United States of America

## Abstract

Malate dehydrogenase (MDH) catalyzes the conversion of oxaloacetate and malate by using the NAD/NADH coenzyme system. The system is used as a conjugate for enzyme immunoassays of a wide variety of compounds, such as illegal drugs, drugs used in therapeutic applications and hormones. We elucidated the biochemical and structural features of MDH from *Thermus thermophilus* (TtMDH) for use in various biotechnological applications. The biochemical characterization of recombinant TtMDH revealed greatly increased activity above 60°C and specific activity of about 2,600 U/mg with optimal temperature of 90°C. Analysis of crystal structures of apo and NAD-bound forms of TtMDH revealed a slight movement of the binding loop and few structural elements around the co-substrate binding packet in the presence of NAD. The overall structures did not change much and retained all related positions, which agrees with the CD analyses. Further molecular dynamics (MD) simulation at higher temperatures were used to reconstruct structures from the crystal structure of TtMDH. Interestingly, at the simulated structure of 353 K, a large change occurred around the active site such that with increasing temperature, a mobile loop was closed to co-substrate binding region. From biochemical characterization, structural comparison and MD simulations, the thermal-induced conformational change of the co-substrate binding loop of TtMDH may contribute to the essential movement of the enzyme for admitting NAD and may benefit the enzyme's activity.

## Introduction

Malate dehydrogenase (MDH, EC 1.1.1.37) catalyzes a dehydrogenation reaction from malic acid to generate oxaloacetic acid, which is accompanied by reduced NAD generating NADH. MDHs play crucial roles in various cellular processes, so the biochemical and structural properties of these enzymes from eukaryal, bacterial and archaeal sources have been studied extensively [1], [2]. Although MDHs catalyze the reversible conversion from malate to oxaloacetate, they differ in coenzyme specificity, cellular localization, and physiological function. Eukaryotic cells contain two NAD-dependent MDHs, including cytoplasmic and mitochondrial types. Mitochondrial MDH is involved in the tricaboxylic acid cycle [3] and cytoplasmic MDH is responsible for the oxaloacetate–malate shuttle through the mitochondrial membrane in conjunction with the mitochondrial enzyme.

In plants, chloroplastic NADP-dependent MDH is regulated by reduced thioredoxin and plays an important role in CO_2_ fixation in photosynthesis [4]. In contrast, bacterial cells contain a single NAD-dependent MDH, which is similar in amino acid sequence to mitochondrial MDH [5]. In addition, MDHs and lactate dehydrogenases (LDHs) are members of a large protein family that can be divided into three sub-families [6]: tetrameric LDH, dimeric MDH and [LDH-like] MDH, whose primary and quaternary structures are similar to those of LDH.

*Thermus thermophilus* is a Gram-negative eubacterium that is extremely thermophilic, with optimal growth temperature of about 65°C. Its genome was originally sequenced and annotated in 2004 [7]. In general, thermophilic enzymes are optimally active at the high temperatures at which their host organisms thrive and are relatively inactive at low temperatures. MDH from *T. thermophilus* (TtMDH) is more similar in amino acid sequence to NADP-dependent chloroplastic MDH than NAD-dependent bacterial and mitochondrial MDHs.

MDH is used in biotechnological applications as a conjugate for enzyme immunoassay of a wide variety of compounds, including illegal drugs, drugs used in therapeutic applications, and hormones [8]. Thus, enzymes with high enzymatic efficiency combined with high protein stability are desired. Although numerous thermophilic enzymes have been characterized, the molecular basis of their action mode at high temperature is still poorly understood. To gain insights into the structural determinants of the thermal-induced activity, we performed a functional and structural characterization of the hyperthermostable MDH from *T. thermophilus*. Results from crystallographic and molecular dynamics (MD) studies along with biochemical characterization revealed important issues in the thermal-induced activity of TtMDH with the modulation of its action at high temperatures by conformational changes in the co-substrate NAD binding area.

## Results and Discussion

### Biochemical properties of TtMDH

**Figure 1:**
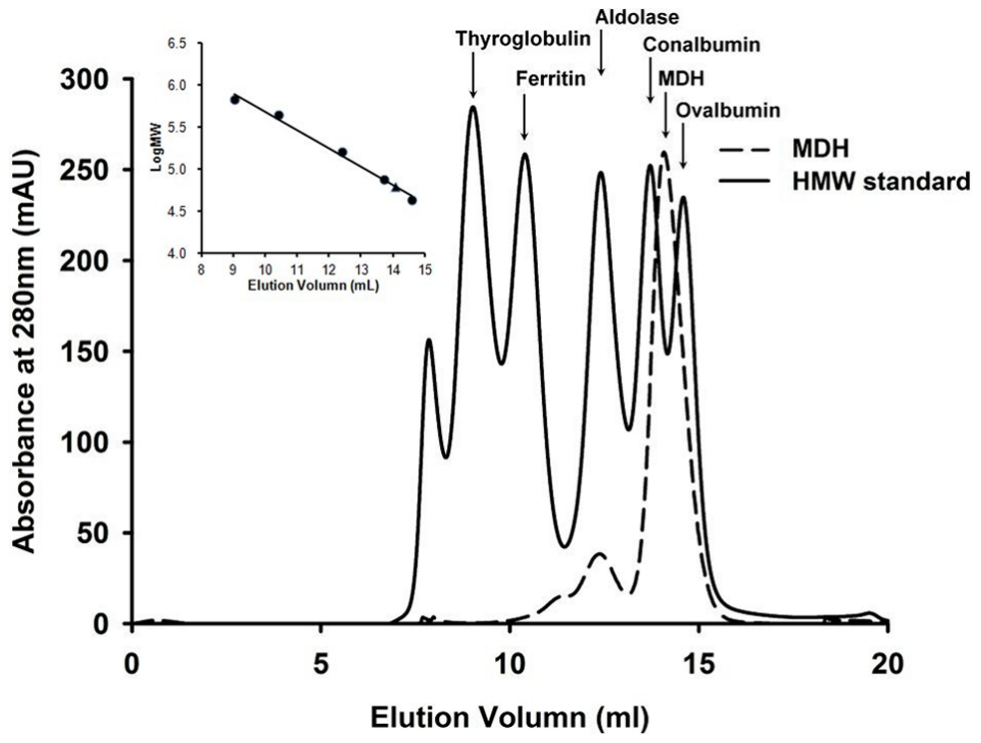
Analytical gel filtration chromatography of *Thermus thermophilus* MDH (TtMDH). TtMDH (0.1 ml with 8.8 mg/ml) was subjected to Superdex 200 HR and the elution profile was compared with high-molecular-weight (HMW) standard proteins (thyroglobulin, 669 kDa; ferritin, 440 kDa; aldolase, 158 kDa; conalbumin, 75 kDa; and ovalbumin, 43 kDa, obtained from GE Healthcare). TtMDH showed a peak with estimated molecular mass 61 kDa. Inset, elution volumes of peaks of HMW standards (•) and peak TtMDH (▴) in the logarithmic scale.

*TtMDH* was amplified from *Thermus thermophilus* chromosomal DNA and cloned into the pET-21b vector (Novagen) to allow for expression of recombinant protein with an additional C-terminal His-tag. After bacterial protein expression and purification, TtMDH protein was then concentrated to 15 mg/ml in 20 mM Tris-HCl (pH 8.0) for the following experiments. We determined the molecular mass of recombinant TtMDH as 37 kDa, calculated from the amino acid sequence (36874.48 Da), and considered similar to that monitored by SDS-PAGE. The molecular mass of TtMDH in solution determined by analytical gel filtration chromatography was about 61 kDa (Figure 1), so TtMDH is a homodimeric enzyme like most MDHs [9].

**Figure 2:**
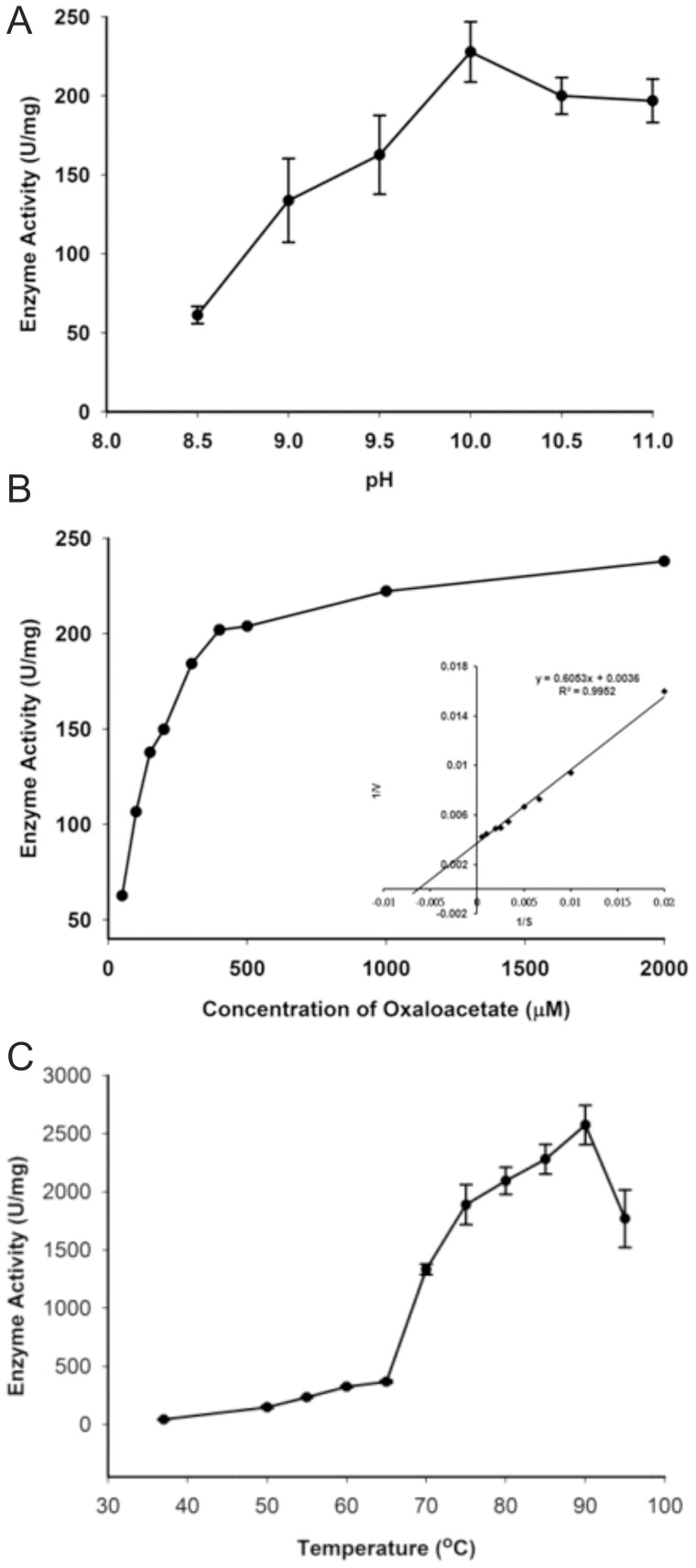
Enzymatic activity of TtMDH. (A) Effect of pH on TtMDH enzymatic activity measured with 100 mM glycine-NaOH buffer of pH 8.5–11.0 under standard assay conditions. All TtMDH activity values were obtained from the means of independent experiments. Specific activity is expressed as units per mg protein; 1 unit is defined as 1 μmole NADH comsumed per minute. (B) Kinetic analysis of TtMDH. The initial enzymatic reaction velocity was measured at 0.15 mM NADH with oxaloacetate concentration from 2–0.015 mM in glycine-NaOH buffer (pH 10.0) at 37°C. The *K*_M_, V_max_ and *k*_cat_ values were calculated from Lineweaver-Burk plots (the double-reciprocal plot showed in the inset). (C) Temperature profile of TtMDH. The optimum temperature for enzyme activity was determined by measuring TtMDH activity at various temperatures from 37°C to 95°C under standard assay condition. All values of TtMDH activity were obtained from the means of three independent experiments. Specific activity is expressed as units per mg protein; 1 unit is defined as 1 μmole NADH consumed per minute.

Of interest, the optimal pH for TtMDH was in the alkaline range. We selected glycine-NaOH buffer with pH 8.5 to 11 to further examine enzyme activity at 37°C. TtMDH showed an increasing of enzyme activity from pH 8.5 to 9.5 and reached maximal activity at pH 10.0 (Figure 2A). In addition, we observed a slight decrease in enzyme activity at pH 10.5 to 11.0 (∼85–90% of maximal activity). This observation was confirmed by repeating the experiment with finer grids around pH 10.0. The pH profile of TtMDH indicated that TtMDH is an alkaline malate dehydrogenase.

We then evaluated the dependence of reaction on substrate concentration for kinetic characterization of TtMDH. The initial velocity against various oxaloacetate concentrations with a fixed concentration of NADH was measured in glycine-NaOH buffer (pH 10.0) at 37°C. The saturation kinetics fit the Michaelis-Menten plot. The data were transformed into a double-reciprocal plot to give a straight line for calculating kinetic parameters (Figure 2B). The apparent K_M_, V_max_, and k_cat_ were 168.1 μM, 277.7 μmole·mg^−1^·min^−1^, and 281.6 s^−1^, respectively.The optimum temperature for enzyme activity was determined by measuring TtMDH activity at various temperatures from 37°C to 95°C under standard assay condition. All values of TtMDH activity were obtained from the means of three independent experiments. The temperature profile showed that TtMDH possessed specific activity of 43 U/mg at 37°C, whereas activity was greatly increased after 60°C and showed the largest specific activity of about 2,600 U/mg at 90°C (Figure 2C).

### Thermal stability of TtMDH

**Figure 3:**
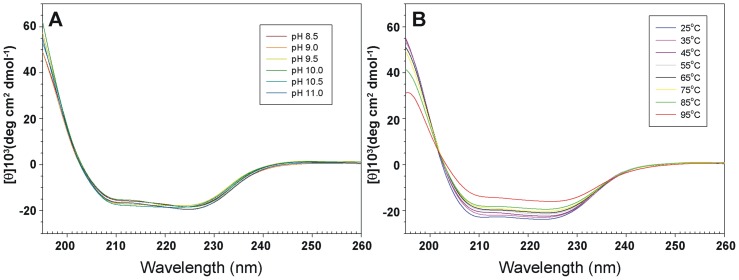
Spectroscopy features and thermal stability of TtMDH. Far-UV circular dichroism (CD) spectra (195–260 nm) for TtMDH at different (A) pH values and (B) temperature. CD spectra were measured from 260–190 nm at different pH values (pH 8.5–11.0) at 25°C and at various temperatures from 25–95°C at pH 7.0.

The biological function of an enzyme is closely related to its overall folding. To determine how TtMDH retains its activity under high pH, we examined its secondary structure at several pHs by CD spectroscopy. Both n→π* and π→π* transitions occurred in the far-UV spectral region (Figure 3A), which corresponds to the electronic transitions within the amide backbone of proteins. TtMDH showed similar CD spectra and contained approximately 41% α-helix and 14% β-strand conformations at various pH ranges (Figure 3A), as calculated by the K2D2 server [10]. Therefore, TtMDH should be active even at high pH values because its secondary structure remained intact. Furthermore, pH tolerance studies showed that TtMDH maintained its intact secondary structure and low but significant catalytic activity.

We investigated the secondary structure of TtMDH under different temperatures at the representative pH 7.0. The far-UV CD spectrum for TtMDH at 25°C showed a characteristic curve of α/β proteins with maximum at 195 nm and minimum at 210 and 225 nm (Figure 3B). CD experiments of TtMDH at different temperatures were shown in similar CD spectral profiles. The secondary structure components of TtMDH contained approximately 40% α-helix and 14% β-sheet conformations over the temperature range tested (Figure 3B). Although the signal for the CD spectrum was lower at 95°C than at 25°C, the findings indicated that a significant amount of TtMDH remained folded in solution at high temperature. Of note, when a protein becomes completely unfolded in solution, the π-bond delocalization is lost along the amide backbone, thus resulting in a CD signal of zero in the far-UV range. Unfortunately, the current setup on the CD spectrometer limits measurements to <100°C. We could not determine the Tm value of TtMDH because it did not reach a completely denatured state even at near 100°C.

### Structure of apo TtMDH

**Figure 4:**
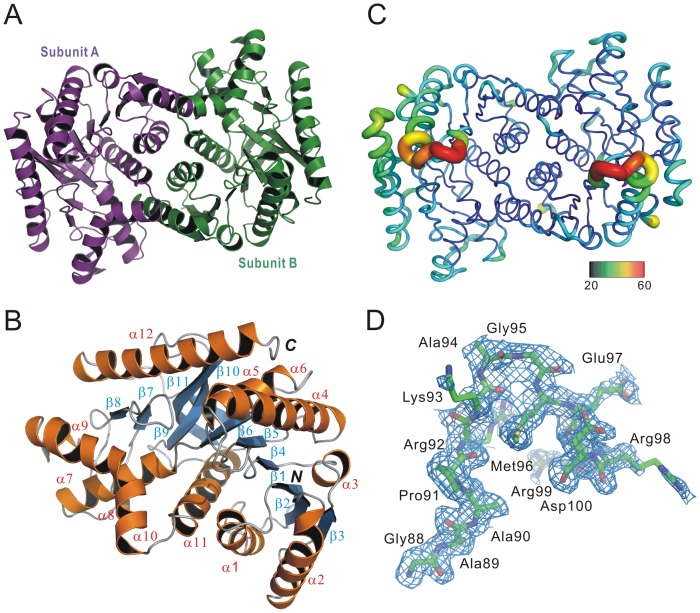
Crystal structure of TtMDH. (A) Ribbon drawing shows 2 monomers related by a pseudo-two-fold. Each monomer consists of 12 helices and 11 strands. (B) Subunit A structure of TtMDH shown as a ribbon representation by 90° rotation around the Z-axis. Secondary structure elements are numbered sequentially as α1–α12 and β1–β11 for the common α-helices and β-strands, respectively. *N* and *C* refer to N- and C-terminal regions, respectively. (C) Sausage representation colored by B-factor. The color range bar represents the B-factor scale from 20 (blue) to 60 (orange) Å^2^. (D) Representative 2*F_o_*-*F_c_* electron-density maps contoured at 1σ.

To understand in detail the structural basis for enzyme catalytic properties, we crystallized the recombinant TtMDH for structure determination with gel filtration chromatography as the final step of TtMDH purification. The crystal structure of TtMDH in the apo form, belonging to the orthorhombic *P*2_1_2_1_2_1_ space group, was refined to resolution 1.80 Å (*R*_work_  = 14.40%, *R*_free_  = 16.98%) with high-quality backbone geometry (Figure S1). The x-ray diffraction data and refinement statistics are in Table 1. The crystallographic asymmetric unit contains two subunits, which forms a homodimer arranged through a noncrystallographic two-fold axis (Figure 4A) and is consistent with the observation of analytical gel filtration chromatography. The two subunits are virtually superimposable, with a root mean square deviation (RMSD) of 0.124 Å between the corresponding Cα atoms of the two subunits. Similar to other MDHs, a protomer of TtMDH has the N-terminal NAD binding domain and C-terminal catalytic domain and consists of 12 helices and 11 β-strands (Figure 4B). The N-terminal NAD binding domain (residue 1–156) is an open twisted structure with the classical Rossmann fold composed of a parallel six-stranded β-sheet (β1–β6) and four α-helices (α1–α4). The C-terminal catalytic domain comprises an antiparallel twisted five-stranded sheet (β7–β11) surrounded by eight α-helices (α5–α12). Two monomers are further assembled into a dimer via reciprocal interactions from helices α1, α2, α7, α8, α9, α10, and α11. The dimeric interface is about 1619 Å^2^ and involves 18 hydrogen bonds and extensive hydrophobic interaction(Figure S2), as analyzed by PDBsum [11]. The overall B-factor of the crystal structure color coded as defined in Figure 4C is quite low, which indicates the high stability of TtMDH. Conversely, the loop region between β4 and α4 reveals higher B-factor values, considered to be involved in conformational changes with NAD binding. Of note, this region in cytoplasmic MDH is disordered [12] and in LDH undergoes a significant conformational change with substrate binding [13]. However, electron densities in the apo-form crystals were of high quality and generally continuous, so the residues of the entire sequence could be modeled into the structure. Even the electron densities of the loop regions for NAD binding are clearly shown (Figure 4D). For most MDHs, the relatively loop close to the substrate binding site is flexible and not well defined in any of the structures.

**Table 1:** Data collection and refinement statistics for apo and NAD-bound forms of *Thermus thermophilus* MDH (TtMDH).

Crystal	apo TtMDH	NAD-bound TtMDH
Space group	P2_1_2_1_2_1_	P2_1_2_1_2_1_
PDB code	4KDE	4KDF
Unit cell parameters		
a, b, c (Å)	71.3; 86.1; 118.2	96.6; 114.6; 144.1
α,β,γ(°)	90, 90, 90	90, 90, 90
Resolution range (Å)	27.86–1.80 (1.86–1.80)	24.27–2.36 (2.44–2.36)
Unique reflections^a^	68001 (6665)	66054 (6067)
〈I〉/〈σ〉^a^	27.7 (6.1)	12.8 (6.3)
R_merge_ a,b (%)	6.1 (30.1)	1.9 (20.4)
Completeness^a^ (%)	99.9 (99.2)	99.0 (92.1)
Redundancy^a^	6.0 (6.0)	7.8 (7.1)
Wilson B-factor	15.02	35.31
Refinement		
Resolution range (Å)	27.86–1.80	24.27–2.36
Reflections (work/test)	81704/4325	59831/3193
R (%)/R_free_ (%)^c^	14.40/16.98	18.24/22.63
No. of water molecules	846	254
Ramachandran plot (%)^d^	92.5/7.5/0/0	93.6/6.3/0.1/0

^a^ Values in parentheses are for the highest resolution shell.

^b^ R_merge_  = Σ_hkl_Σ_i_|I_i_(hkl)−〈I_i_(hkl)〉|/Σ_hkl_Σ_i_I_i_(hkl), where I_i_(hkl) is the intensity of the i_th_ observation and 〈I(hkl)〉 is the mean intensity of the reflections.

^c^ R/R_free_  = Σ_hkl_||Fobs| − |F_calc_||/Σ_hkl_|F_obs_|, where R and R_free_ are calculated by the working and test reflections, respectively.

^d^ Percentage of residues in most favoured/additionally allowed/generously allowed/disallowed regions of Ramachandran plot, according to PROCHECK [34].

### Structure of NAD-bound TtMDH

To gain further insight into the co-substrate recognition and activity relationship for TtMDH, we attempted to soak the apo-form crystal with NAD. The electron density maps obtained throughout the structure determination process were of high quality and allowed for easy model reconstruction and refinement. Nevertheless, electron densities for residues 91–100 were relatively weak and poorly defined. They correspond to the mobile loop covering the catalytic site, which is disordered as in the 3D structures of most other MDHs [14], [15]. In addition, we found a mass of electron density in co-substrate binding site of TtMDH that could be modeled as NAD (Figure S3). Unlike the apo form of TtMDH, crystal structure of NAD-bound TtMDH belongs to the *P*2_1_2_1_2_1_ space group with four molecules in an asymmetric unit (Table 1); however, a homodimer should be formed with identical subunits in the crystal lattice. The NAD-bound structure was refined to 2.36 Å (*R*_work_  = 18.24%, *R*_free_  = 22.63%) with good stereochemistry (Figure S4). NAD binds to TtMDH in the same extended conformation seen for other members of this enzyme family [1], [16]. The adenine ring binds in a hydrophobic crevice defined by Leu41, Ile43, Ile108, Thr10, Gly11, Gly88, and the backbone of Gly88 and Ala89. Two hydrogen bonds were observed for Gln112 and Gly11, respectively, binding to the adenine ring nitrogen atoms (Figure S3). Residue 42 of TtMDH is a glutamate, which hydrogen-bonds to the adenine ribose instead of the otherwise conserved aspartate in other MDHs [12], [17]. The NAD pyrophosphate is hydrogen-bonded to the backbone of residue 15 and 16, but nicotinamide ribose is hydrogen-bonded to the side-chain of residue Asn131 through 2′-hydroxyl. These interactions are virtually identical to those found in cytoplasmic MDH.

To elucidate the conformational change of a loop with NAD binding, we compared the overall structures of apo and NAD-bound TtMDH. The overall structures in the absence or presence of NAD were similar, with RMSD 0.254 Å for the main chain. Both structures present good overall stereochemistry (Table 1) with small RMSD between apo and NAD-bound structures, which suggests that the presence of NAD did not greatly alter the protein conformation and, consequently, the crystal packing contacts. However, the increasing B-factor value, slight movement of the binding loop and few structural elements around the NAD binding site indicate that conformational change is significant for TtMDH activity.

### MD simulation of TtMDH

Biochemical characterization of recombinant TtMDH showed greatly increased activity above 60°C and the greatest specific activity with optimal temperature 90°C. However, the mechanism of thermal-induced TtMDH activity remained to be investigated. Crystal structures of apo and NAD-bound forms of TtMDH revealed slight movement of the binding loop and a few structural elements around the binding packet in the presence of NAD. The motion of the binding loop may play a key role in NAD binding for the enzyme activity, and increasing temperature may contribute to the necessary breathing motion of TtMDH for the NAD binding packet. To verify this hypothesis, we used MD simulation to explore the dynamic behavior of TtMDH at different temperatures.

**Figure 5:**
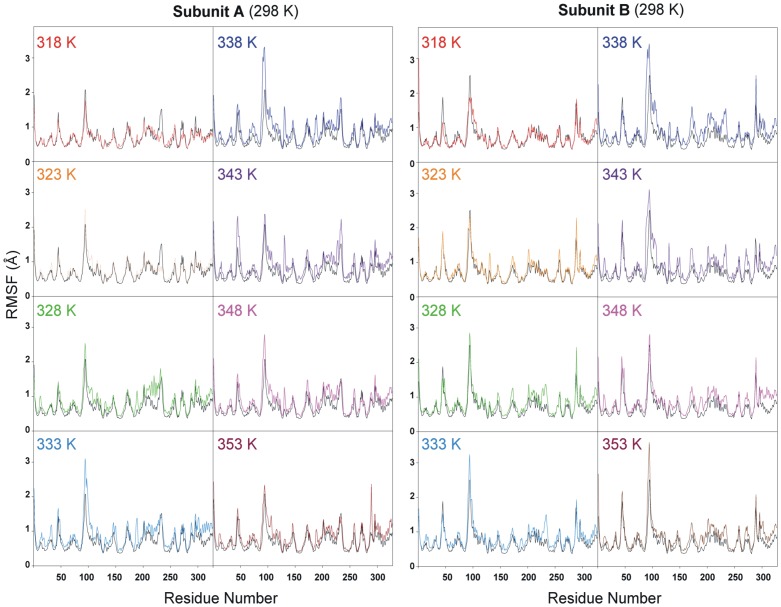
Root mean square fluctuation of C^α^ atoms of TtMDH after fitting the corresponding atom positions from molecular dynamics (MD) trajectory to the initial (X-ray) coordinates. Results from different trajectories (different temperatures) are indicated with different-colored lines and compared with the trajectory at 298 K (black line).

We performed nine MD simulations at various temperature values (298, 318, 323, 328, 333, 338, 343, 348, and 353 K). All MD simulations were performed with GROMOS96 forcefield with GROMACS 4.5.3 package running on a high-performance Linux cluster computer. The MD protocol was based on previous studies [18] and evaluated the TtMDH structure at every 5 degrees from 318 to 353 K for 10 ns. Trajectories over 10 ns were analyzed by calculating the RMSD of apo form TtMDH crystal structure with the simulated structures at 298 K and other temperatures (Figure S5). At the beginning of the trajectory (t = 0), the RMSD for all sets of MDHs was 0.8 Å, which indicated that the movement of all sets of MDHs occurred during the thermalization and equilibration periods. Thus, the TtMDH structure was stable and could maintain the conformation at even 353 K. We used root mean square fluctuation (RMSF) of each residue in TtMDH to calculate over the trajectory for overall flexibility of the systems at different temperatures. At 318 to 353 K, TtMDH showed a slight fluctuation with amino acids 90–110, the binding loop region of TtMDH (Figure 5A). This region consists of partially an α-helix and a coil located in the surface of the TtMDH structure. As shown in Figure 4C, this binding loop region between β4 and α4 of TtMDH reveals higher B-factor (around 0.5) consistently. In contrast, other loop regions of TtMDH with much lower B-factor (around 0.2), exhibits only little fluctuation of simulated structures at high temperatures. The fluctuation of the binding loop region of TtMDH then increased the flexibility at higher temperature (353 K; Figure 5B) indicated that the binding loop was a thermal-sensitive region.

**Figure 6:**
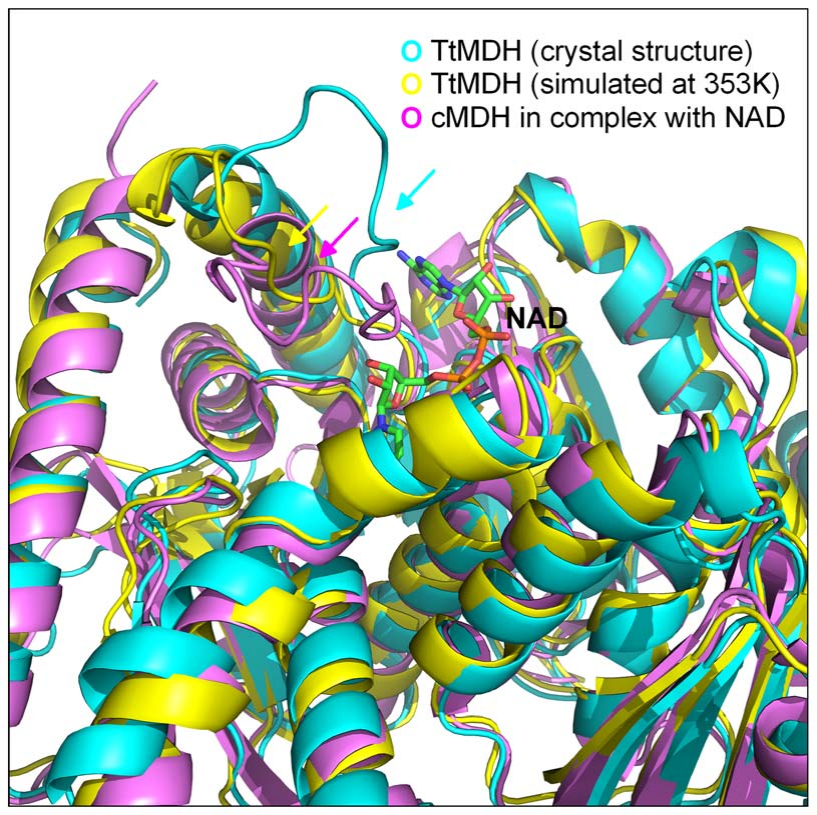
Ribbon diagrams of crystallographic and high-temperature–simulated TtMDH as well as cytoplasmic MDH (cMDH) in complex with NAD showing the structural similarity of the enzymes and the large conformational differences of the co-substrate NAD binding loop. For TtMDH (cyan) the loop region is open, whereas for cMDH (pink), the loop adopts a compact conformation. For the high-temperature–simulated TtMDH, the loop is in a more closed position as for cMDH.

As compared with the crystal structure, the final frame of TtMDH structure from MD simulations at 80°C (353 K) showed a significant difference only around the product release area without disturbing the overall fold and structure (Figure 6). At 80°C, the binding loop comprising residues 90–110 was displaced far away from its original conformation, thus resulting in a marked loop motion around the catalytic pocket and tuning the loop position for NADH binding. Superposition of the crystal structure and high-temperature–simulated MDHs with a cytoplasmic MDH (cMDH) complex revealed a large change around the NADH binding site. In the cMDH–NAD complex, the mobile loop closed to bring key residues into contact with the co-substrate, but the crystal structure of TtMDH presents an open conformation of the binding loop. Interestingly, the simulated TtMDH structure at high temperature has the loop in a more closed position to adopt a compact conformation. Thus, the critical increasing temperature may improve the flexibility of the binding loop region for better plasticity of the co-substrate binding pocket of TtMDH for NAD entry.

### NAD-binding affinity of TtMDH

We investigated the thermal-induced activity associated with critical loop movement for NAD binding by measuring the binding properties for different temperatures. Thus, ITC measurements were used to accurately determine the binding parameter and characterizations of TtMDH (Figure S6). The dissociation constant *K*_d_ for NAD to TtMDH at 298 and 353 K were 1.16 and 0.263 μM, respectively (Table 2). Normally, binding affinities for molecular interaction is decreased at higher temperature because of reduced formation of intermolecular non-covalent bonding. Therefore, TtMDH has better NAD binding affinity at higher temperature, which is in good agreement with the optimal temperature for the greatest enzymatic activity. The thermal-induced activity of TtMDH was induced by breakage of intramolecular non-covalent bonds and increased flexibility of the binding pocket for co-substrate entry or binding.

**Table 2:** Thermodynamic parameters for NADH binding to TtMDH.

Ligand	T	ΔH (KJ mol^−1^)	ΔS (J mol^−1^ K^−1^)	ΔG (KJ mol^−1^)	N	*K*_d_ (μM)
NADH	298 K	−6.47	91.9	−33.86	1.04	1.160
NADH	353 K	5.05	140.3	−44.48	1.03	0.263

## Conclusions

MDHs can be classified into NAD-dependent MDHs (NAD-MDHs) and NADP-dependent MDHs (NADP-MDHs). Most bacterial and archaeal MDHs are NAD-MDHs. NAD-MDH are generally assembled as dimmers or tetramers with subunit molecular mass 30 to 37 kDa [16], [19], [20]. MDHs found in *Thermus flavus* [21], *Thermus aquaticus* [22], *Acetobacter* sp. [23], *Streptomyces coelicolor* [20] and higher plant cytosols [19] have a dimeric structure, whereas those in *Bacillus* sp. [17], *Flavobacterium frigidimaris* [24], and *Aeropyrum pernix* [25] have a tetrameric structure. Analytical gel filtration chromatography revealed that MDH from *T. thermophilus* was a dimer, which is consistent with the quaternary structure of the *Thermus* genus [21], [22].

Biochemical characterization showed that TtMDH had specific activity of 34 U/mg at 37°C and the activity increased with increasing temperature of the enzyme assay; however, the activity increased greatly above 60°C and the optimal temperature was 90°C with specific activity about 2,600 U/mg, more than 300-fold activity at 37°C. Stability analysis of TtMDH showed that it was highly stable because it still retained almost full activity with heat treatment for 30 min at different temperatures <90°C. CD experiments revealed high stability, with similar spectra at different temperatures and pH values. In addition, TtMDH crystals grew and diffracted to high resolution in the presence and absence of co-substrates at 1.80 and 2.36 Å, respectively. However, the structures were similar and could not explain the thermal-induced structural features of the thermophilic enzyme activity of TtMDH. Thus, MD simulation was performed to investigate the possible local conformational change of TtMDH at high temperature for its unique activity.

Crystal structures of apo and NAD-bound forms of TtMDH revealed a slight movement of the binding loop and few structural elements around binding packet in the presence of NAD, so the motion of the binding loop should play a key role in NAD binding for enzyme activity. The increasing temperature may contribute to the necessary binding loop motion of TtMDH for the NAD binding packet. To verify this hypothesis, MD simulation was used to explore the dynamic behavior of TtMDH at different temperatures. The simulated structure showed a few small changes around the active site to make it fit substrates with increasing temperature and resulted in increased activity, but the overall structures did not change much and retained all related positions. This finding agrees with the CD analysis of TtMDH. Our structural comparisons and MD simulations reveal that the thermal-induced conformational change of TtMDH contributes to the essential movement of the enzyme for adapting NAD and benefits the enzyme activity. Our studies shed light on the structure–activity relationship of TtMDH and may help in reducing the optimal temperature for the greatest enzymatic activity for thermostable MDH in biotechnological applications.

## Materials and Methods

### Cloning, protein expression and purification

We used PCR with *T. thermophilus* genomic DNA to amplify the coding sequence of TtMDH, which was cloned into the pET21b vector system (Novagen). Recombinant TtMDH with a hexa-histidine (6 x His) tag at the C terminus was overexpressed in *E. coli* BL21(DE3) with Luria broth culture medium containing 100 μg/ml ampicillin. The cells were grown at 37°C up to OD_600_ 1.0. The expression of recombinant TtMDH was induced with 1 mM isopropyl-β-D-thiogalactoside and cell growth continued for another 2 h at 37°C. Cells were collected by centrifugation and resuspended in lysis buffer (100 mM Tris-HCl, pH 8.0, 150 mM NaCl). After 10 min of sonication, the cell extract was clarified by centrifugation at 12,500 rpm for 30 min at 4°C to remove debris. The supernatant was treated with heat incubation at 70°C for 30 min and centrifuged at 12,500 rpm to remove the undesired denatured proteins. The clear supernatant was then placed in an open column filled with Ni^2+^-NTA resin according to the manual's instruction. By enzyme assay and SDS-PAGE analysis, the desired fractions were pooled. The pooled fractions were concentrated to <0.5 ml and then placed in a column packed with superdex 200 HR (GE Healthcare) for gel filtration chromatography to yield a homogeneous protein and to determine the molecular mass of TtMDH in solution state.

### Biochemical characterization

The activity of TtMDH was determined by monitoring the decrease of absorbance at 340 nm due to consumption of NADH for 3–5 min and then converted to enzymatic activity. The mixture contained 0.2 mM oxaloacetate, 0.15 mM NADH, 100 mM glycine-NaOH buffer (pH 10.0); an appropriate amount of enzyme was prepared in a total volume of 1 ml. The reaction was initiated by adding the enzyme and continued at 37°C. The increase in absorbance at 340 nm was monitored for 2 to 3 min, which represents the consumption of NADH. One unit (U) of TtMDH activity was defined as the amount of enzyme that consumed 1 μmole NADH per minute. Protein concentration was determined by the Bradford method with Bio-Rad protein assay reagent, and bovine serum albumin was used as a standard.

TtMDH activity was screened by Tris-HCl, Bis-Tris–Propane, HEPES and glycine-NaOH buffers. The optimal pH value for TtMDH activity was confirmed by triple repeats in glycine-NaOH buffer with varied pH from 8.5 to 11. Apparent kinetic constants were determined by assaying enzyme activity at various concentrations of oxaloacetic acid ranging from 2 to 0.05 mM with a fixed amount of 0.15 mM NADH in glycine-NaOH buffer (pH 10.0) at 37°C. The change in absorbance at 340 nm was recorded between 10 to 30 s. Data were calculated by Michaelis-Menten and Lineweaver-Burk plots (the double-reciprocal plot). *K*_M_, V_max_, and *k*_cat_ values were calculated by Lineweaver-Burk plots.

### Circular dichroism (CD) spectroscopy

Far-UV CD spectra were recorded on a Jasco J-810 spectropolarimeter (Jasco International Co.). CD measurements were carried out in a 1 mm quartz cuvette at wavelength 190 to 260 nm. The protein samples in phosphate buffer were set to 3.2 μM at various pH values from pH 4–8. All samples were centrifuged at 10,000 g for 10 min before analysis. To study the effect of temperature on TtMDH structure, the sample was heated from 20 to 95°C and CD spectra at different temperatures were obtained. The data collection parameters were scan rate 50 nm/min; response time 4 s; sensitivity 100 mdeg; accumulation 6; heating rate 1°C/min; and delay time for spectrum collection 60 s. The reversibility of the temperature effect was checked by cooling the sample to 20°C with the same values. Baseline subtraction, smoothing and data normalization involved use of SigmaPlot. The CD data are shown as mean residue ellipticity units (deg cm^2^ dmol^−1^).

### Crystal structure determination

Crystallization involved a vapor diffusion method with a HoneyBee 963 robot (Genomic Solutions). Sitting drops were prepared by mixing 0.5 μL TtMDH at 10 mg/mL with an equal volume of mother liquor and were equilibrated against 100 μL of the solution at 10°C. Apo-form crystals were obtained in the drop containing 100 mM Tris-HCl, pH 8.2, 22.5%(W/V) PEG4000, and 200 mM magnesium chloride, and reached peak size for X-ray diffraction in 5 days [26]. NAD-bound form crystals were obtained by soaking the apo-form crystal with a 10-μL crystallization reservoir containing 2 mM NAD molecule for about 30 min and then immediately mounted for X-ray diffraction. The diffraction image of the apo and NAD-bound forms were recorded in a 100-K nitrogen gas stream with use of BL13B1 and BL13C1 beamlines (National Synchrotron Radiation Research Center, Taiwan). The wavelength used was 1.0 Å and the intensities were recorded in an ADSC Quantum 315r CCD detector. Data were indexed, integrated and scaled by use of HKL2000 [27].

### Structure solution and refinement

The crystal structures of TtMDH were determined by a molecular replacement method with use of BALBES [28] and the atomic coordinates of *Thernus flavus* MDH (PDB entry code: 1BMD, [29]) as the search model. The initial model was refined by use of the maximum likelihood method implemented in REFMAC5 [30] as part of the CCP4 suite [31] and rebuilt interactively based on inspection of the σ-weighted electron density maps with coefficients 2*mF*_o_-*DF*_c_ and *mF*_o_-*DF*_c_ with the program COOT [32]. During the later stage, restrained positional and B-factor refinement were performed with the program phenix.refine [33], and non-crystallographic symmetry (NCS) restraints over the two subunits were applied during refinement. Water molecules were manually added at the final stages. The models were evaluated with use of PROCHECK [34] and MOLPROBITY [35]. The final coordinates and structure factors of TtMDH in apo form and NAD-bound form were deposited in the PDB under accession codes 4KDE and 4KDF, respectively. The data collection and structure refinement statistics are in Table 1.

### MD simulations

The TtMDH crystal structure underwent MD simulations to evaluate the thermal-induced conformational change of the enzyme at various temperatures with use of GROMACS 4.5.3 and GROMOS96 43a1 forcefield [37]. During MD simulations, all protein atoms were surrounded by a cubic water box of SPC3 molecules that extended 10 Å from the protein, and periodic boundary conditions were applied in all directions. The systems were neutralized with Na+ and Cl- counter ions replacing the water molecules. Energy minimization involved the steepest descent algorism for 10,000 steps without position restraints. Then the systems underwent equilibration by two steps. The first step involved an NVT ensemble (constant number of particles, volume, and temperature) for 500 ps, and the second step, an NPT ensemble (constant number of particles, pressure, and temperature) for 500 ps. Position restraints were imposed on protein atoms during equilibration to ensure balanced water molecules around the protein. Then, 100-ps position-restrained MD simulations were performed, followed by 10-ns production MD simulations with time step of 2 fs at constant pressure (1 atm) and several temperatures (298, 318, 323, 328, 333, 338, 343, 348, and 353 K). The electrostatic interactions were calculated by the PME algorithm and all bonds were constrained by use of the LINCS algorithm. A twin range cutoff used for long-range interactions was 0.9 nm for van der Waals and 1.4 nm for electrostatic interactions [36]. In this method, short-range interactions are calculated at each step of the simulation, whereas interactions at longer distance are only calculated at each update of the non-bonded pair list and kept constant up to the next update. The reference temperature for coupling was controlled via v-rescale coupling algorithm, and a pressure of 1 atm was maintained by the Parrinello-Rahman algorithm. Snapshots were collected every 1 ps and stored for analysis of MD simulations. The system stability and behavior of the catalytic structural components present in every system were analyzed with use of the tools available in GROMACS 4.5.3 program [37].

### Isothermal titration calorimetry (ITC)

Binding of NADH to TtMDH at different temperatures was measured by ITC with a Nano ITC Isothermal Titration Calorimeter (TA Instruments). Aliquots of 5 μL of 10 mM polyamine were titrated by injection into protein (0.5 mM in 1.3 mL) in 20 mM Tris-HCl (pH 8.0) and 100 mM NaCl at 298 or 353 K. Background heat from ligand to buffer titrations were subtracted, and the corrected heats from the binding reaction were used to derive values for the stoichiometry of the binding (n), association constant (Ka), dissociation constant (Kd), apparent enthalpy of binding (ΔH), entropy change (ΔS), and change in Gibbs free energy that occurred on binding (ΔG = ΔH-TΔS). Data were fitted by use of an independent binding model with Launch NanoAnalyze v2.3.6.

## Supporting Information

Figure S1 **Ramachandran plot for the crystal structure of TtMDH in apo form.** The main-chain torsional angle Phi (N-Cα bond) is plotted against Psi (Cα-C' bond). Blue squares represent non-glycine residues and blue triangles glycine residues. The plot includes both subunits of the dimer.
(TIF)

Figure S2 **Residues involved in dimeric interface of TtMDH.**
(TIF)

Figure S3 **The co-substrate binding site of TtMDH.** (A) The final omit electron density map is overlaid as mesh, contoured at the 1.0σ level. (B) Ligplot diagram of the non-covalent bondings between NAD and the binding pocket of TtMDH.
(TIF)

Figure S4 **Ramachandran plot for the crystal structure of TtMDH in NAD-bound form.** The main-chain torsional angle Phi (N-Cα bond) is plotted against Psi (Cα-C' bond). Blue squares represent non-glycine residues and blue triangles glycine residues. The plot includes four protomers in the asymmetric unit.
(TIF)

Figure S5 **Root mean square deviations in Cα positions for the crystal structure of TtMDH in different protein systems.** The colors for the systems are 318 K, black; 323 K, red; 328 K, green; 333 K, yellow; 338 K, blue; 343 K, pink; 348 K cyan; 353 K grey.
(TIF)

Figure S6 **Isothermal titration calorimetry (ITC) binding curves for the TtMDH complex in combination with NAD at (A) 298 K and (B) 353 K.**
(TIF)
