# Supplementary figures and images for: Crystal Structures and Molecular Dynamics Simulations of Thermophilic Malate Dehydrogenase Reveal Critical Loop Motion for Co-Substrate Binding
Source: PLoS One. 2013 Dec 26;8(12):e83091. doi: 10.1371/journal.pone.0083091 (PMC3873296; doi:10.1371/journal.pone.0083091)

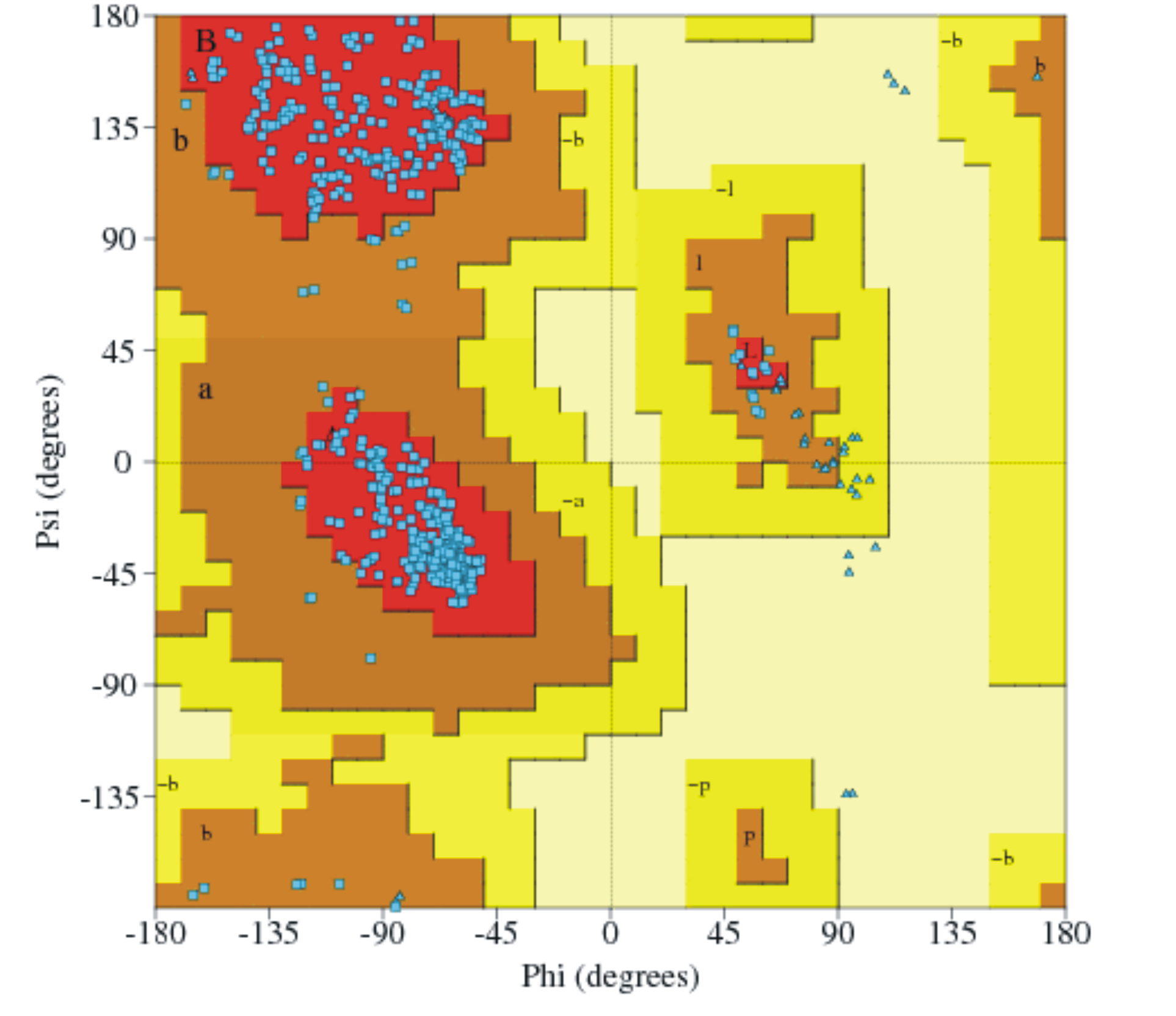

Supplement: Figure S1 — Ramachandran plot for the crystal structure of TtMDH in apo form. The main-chain torsional angle Phi (N-Cα bond) is plotted against Psi (Cα-C' bond). Blue squares represent non-glycine residues and blue triangles glycine residues. The plot includes both subunits of the dimer. (TIF) [file pone.0083091.s001.tif]

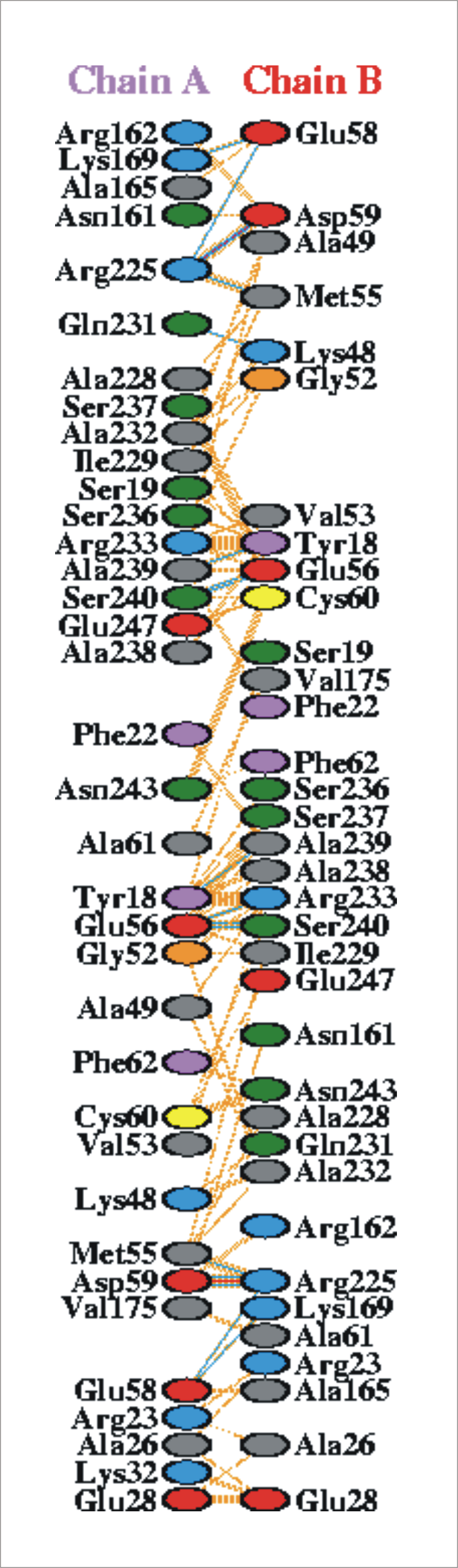

Supplement: Figure S2 — Residues involved in dimeric interface of TtMDH. (TIF) [file pone.0083091.s002.tif]

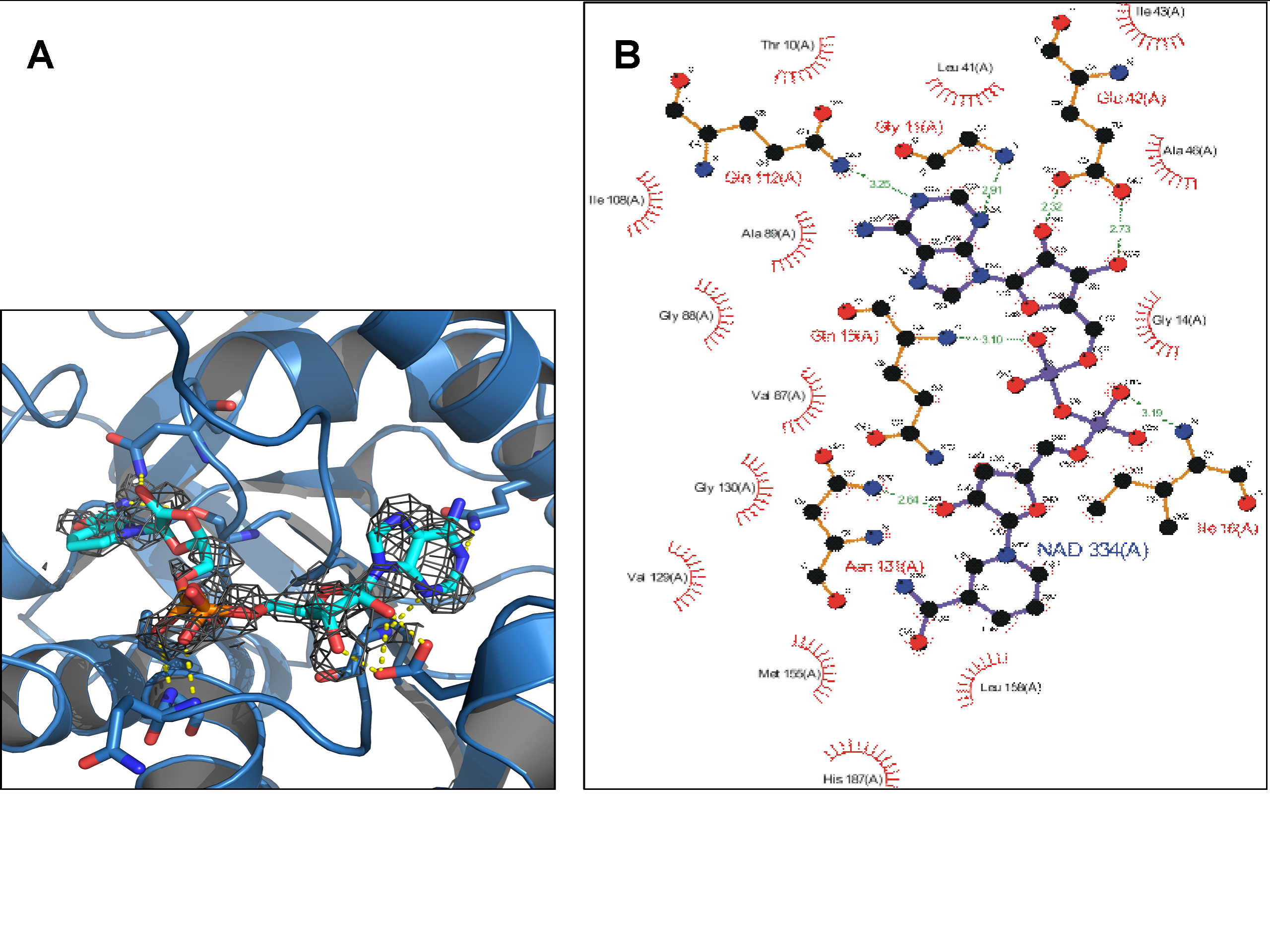

Supplement: Figure S3 — The co-substrate binding site of TtMDH. (A) The final omit electron density map is overlaid as mesh, contoured at the 1.0σ level. (B) Ligplot diagram of the non-covalent bondings between NAD and the binding pocket of TtMDH. (TIF) [file pone.0083091.s003.tif]

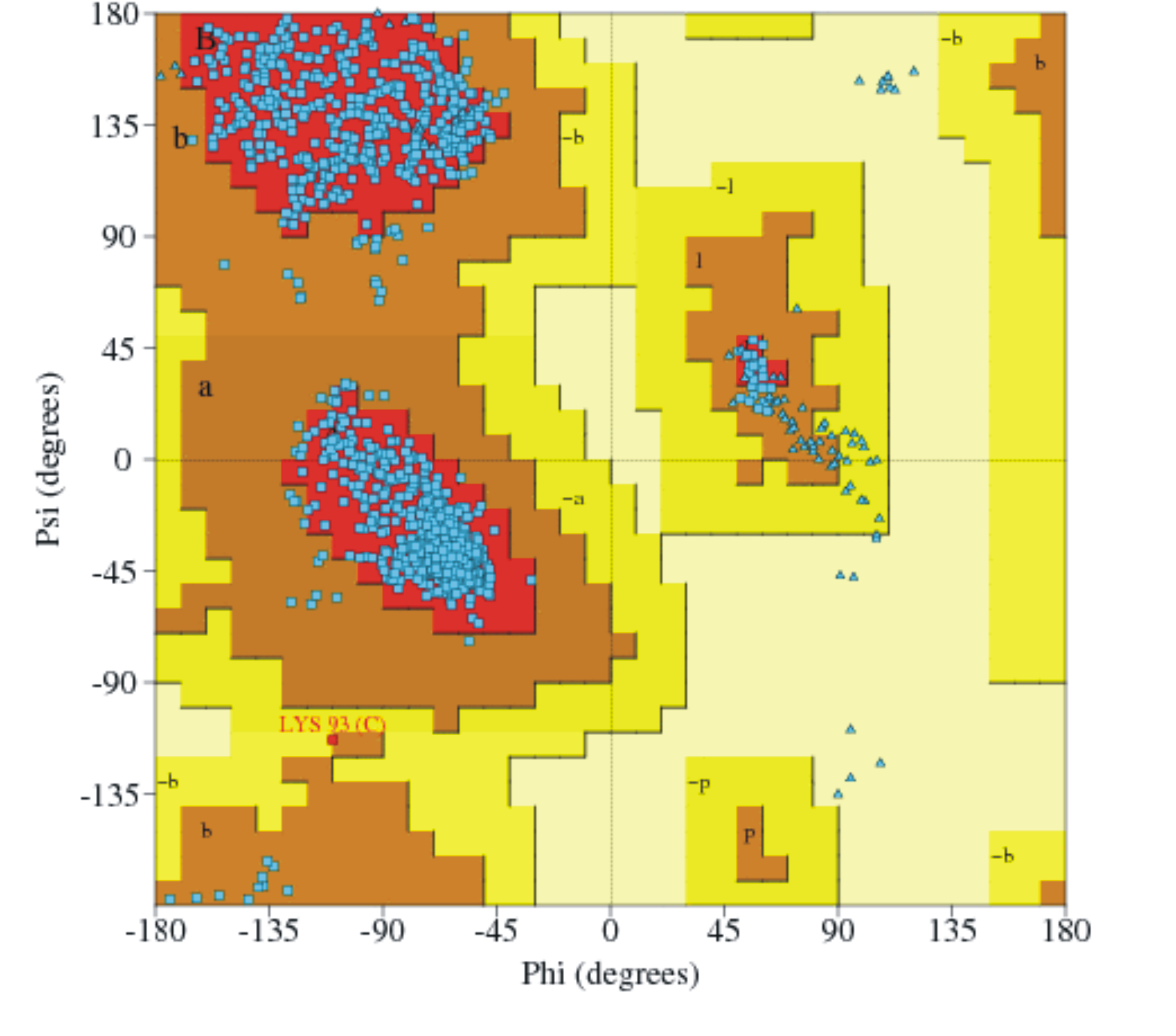

Supplement: Figure S4 — Ramachandran plot for the crystal structure of TtMDH in NAD-bound form. The main-chain torsional angle Phi (N-Cα bond) is plotted against Psi (Cα-C' bond). Blue squares represent non-glycine residues and blue triangles glycine residues. The plot includes four protomers in the asymmetric unit. (TIF) [file pone.0083091.s004.tif]

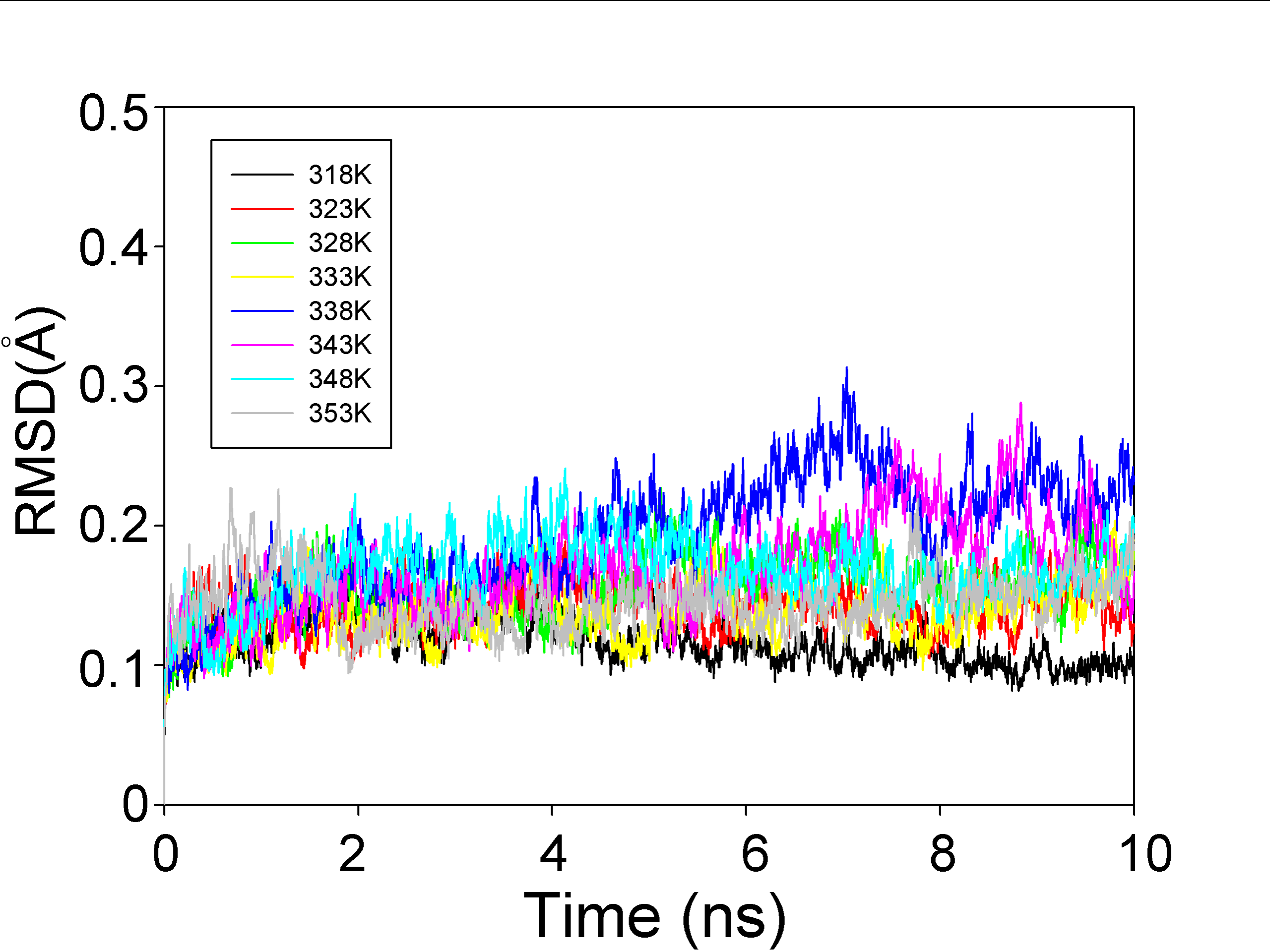

Supplement: Figure S5 — Root mean square deviations in Cα positions for the crystal structure of TtMDH in different protein systems. The colors for the systems are 318 K, black; 323 K, red; 328 K, green; 333 K, yellow; 338 K, blue; 343 K, pink; 348 K cyan; 353 K grey. (TIF) [file pone.0083091.s005.tif]

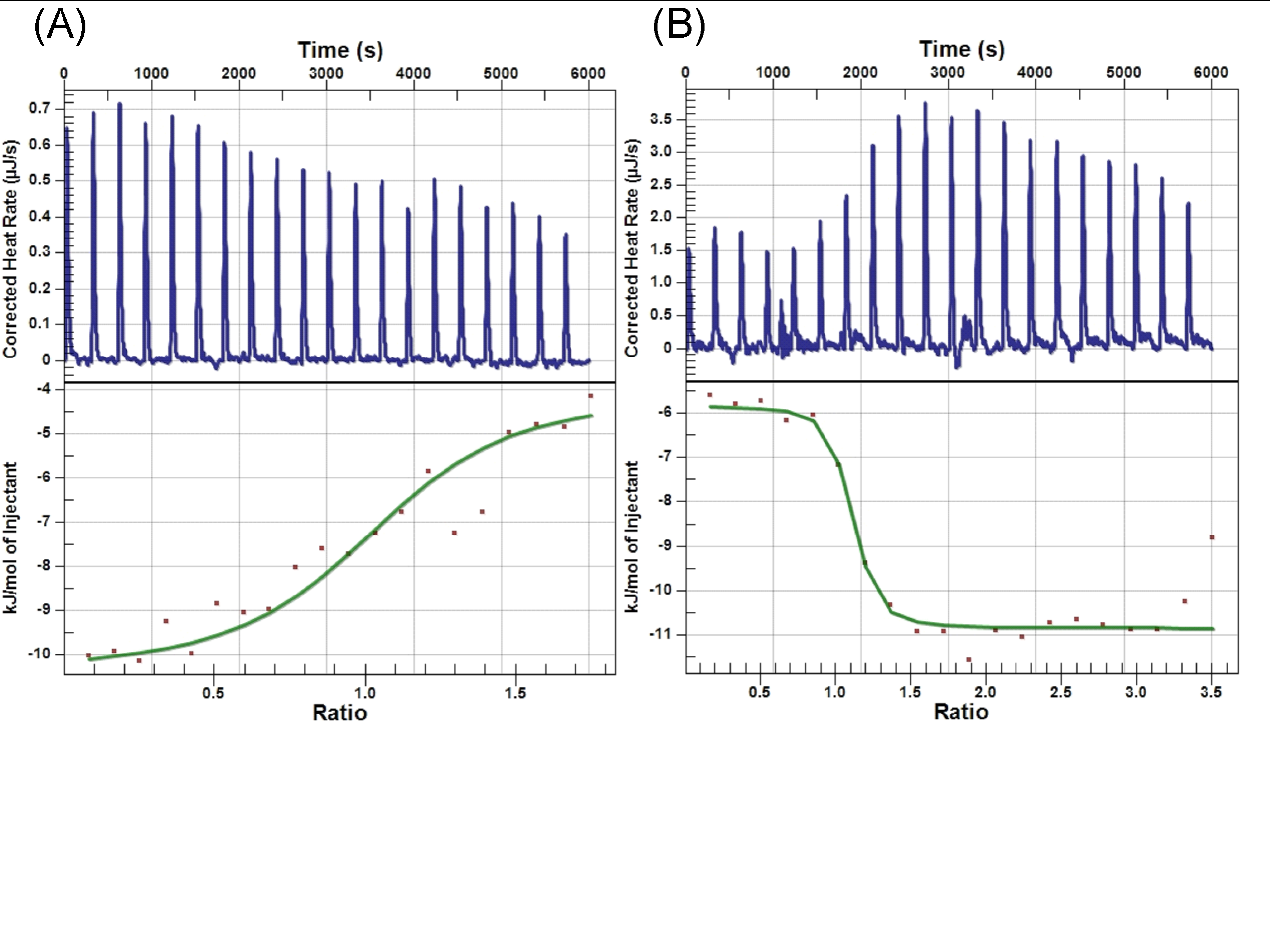

Supplement: Figure S6 — Isothermal titration calorimetry (ITC) binding curves for the TtMDH complex in combination with NAD at (A) 298 K and (B) 353 K. (TIF) [file pone.0083091.s006.tif]
